# Impact of Sjogren's syndrome on Parkinson’s disease: A nationwide case-control study

**DOI:** 10.1371/journal.pone.0175836

**Published:** 2017-07-13

**Authors:** Ming-Chi Wu, Xun Xu, Shan-Ming Chen, Yeu-Sheng Tyan, Jeng-Yuan Chiou, Yu-Hsun Wang, Li-Chi Lin, Chyong-Mei Chen, James Cheng-Chung Wei

**Affiliations:** 1 School of Medicine, Chung Shan Medical University, Taichung, Taiwan; 2 Department of Medical Imaging, Chung Shan Medical University Hospital, Taichung, Taiwan; 3 Department of Medical Informatics, Chung Shan Medical University, Taichung, Taiwan; 4 Department of Radiology, The First Affiliated Hospital with Nanjing Medical University, Nanjing, China; 5 Department of Pediatrics, Chung Shan Medical University Hospital, Taichung, Taiwan; 6 School of Medical Imaging and Radiological Sciences, Chung Shan Medical University, Taichung, Taiwan; 7 School of Health Policy and Management, Chung Shan Medical University, Taichung, Taiwan; 8 Department of Medical Research, Chung Shan Medical University Hospital, Taichung, Taiwan; 9 Division of Allergy, Immunology and Rheumatology, Chung Shan Medical University Hospital; Taichung, Taiwan; 10 Department of Statistics, Oklahoma state University, Stillwater, United States of America; 11 Institute of Public Health, National Yang-Ming University, Taipei, Taiwan; 12 School of Medicine, National Yang-Ming University, Taipei, Taiwan; 13 Institute of Medicine, Chung Shan Medical University, Taichung, Taiwan; Penn State University, UNITED STATES

## Abstract

**Objective:**

To investigate whether Sjogren’s syndrome would have an influence on the development of Parkinson’s disease.

**Methods:**

A population-based case-control study was conducted. Participants consisted of 7716 subjects with newly diagnosed Parkinson’s disease and a population of 75129 matched control subjects between 2000 and 2010. We measured the risk of Parkinson’s disease in association with Sjogren’s syndrome by using adjusted odds ratios.

**Results:**

A total of 143 Parkinson’s disease subjects (1.9%) and 893 control subjects (1.2%) suffered from Sjogren’s syndrome (p < 0.001). The crude odds ratio for Parkinson’s disease among subjects with Sjogren’s syndrome was 1.56 (95% CI 1.30–1.86; p < 0.01). After adjustment for potential confounders which have been proposed that would increase the risk of development of Parkinson’s disease, Sjogren’s syndrome was found to be significantly associated with the risk of Parkinson’s disease with an odds ratio of 1.37 (95% CI 1.15–1.65; p < 0.01).

**Conclusion:**

This study preliminarily proposed that Sjogren’s syndrome was significant associated with an increased risk of Parkinson’s disease.

## Introduction

Parkinson’s disease (PD), known as a common neurodegenerative disease, impacts on people in middle to older age group, and is characterized by rigidity, bradykinesia, resting tremor and postural instability [1–3]. The prevalence of PD per 100,000 of population is 84.8 to 147.7 and the incidence of PD is 28.8 to 35.3 of 100,000 per year in Taiwan[4]. With advance of the disease, various problems, such as cognitive dysfunction and living barrier, are present to disturb the PD patients and their families, which may increase the burden of society and health care system [5,6]. Thus, for the purpose of preventing and early detecting PD, investigation of risk factors for PD is becoming an important study issue nowadays.

As pervious studies revealed, patients with diabetes, hypertension, end-stage renal disease and dyslipidemia would tend to increase the risk of PD [7–9]. Moreover, atrial fibrillation has also been proven to be related to the generation of PD [10]. It was mentioned by some studies that autoimmune diseases, for instance rheumatoid arthritis (RA), systemic lupus erythematosus (SLE), psoriasis, osteoarthritis and vasculitis, were well-recognized risk factors of PD because of the neurologic abnormalities caused by immune damage [11–16]. In addition to these autoimmune factors, few studies introduced that Sjogren’s syndrome (SS) was connected with PD nowadays [17,18].

SS is an autoimmune disease which can enroll multisystem and cause impairment of exocrine glands, leading to dry eyes, dry mouth and salivary gland enlargement. The prevalence of SS is 43.69 to 77.94 per 100 000.The incidence of SS was 4.98 to 8.86 of 100,000 per year in Taiwan [19]. Although the definite cause of SS remains obscure, previous study proposed that lymphocytic infiltration and immunologically mediated mechanisms were connected with the occurrence of SS [20]. When the lymphocytes and immunoreactive proteins infiltrate the nervous systems, some neurological impairment would appear. According to some researches, approximately 31% patients with SS would suffer from the central nervous system(CNS) impairment [21].

Although several studies have investigated the association between SS and PD, the sample sizes of these studies were too small to gain a convincing result [17,18]. Therefore, the purpose of this study is to explore whether SS is associated with the generation of PD by conducting a case-control study based on a nationwide longitudinal population-based database.

## Materials and methods

### 1. Data source

This study used Longitudinal Health Insurance Database (LHID) from 2000 to 2010 which contained 1 million beneficiaries who were randomly sampled from the original National Health Institutes Research Database (NHIRD) beneficiaries in Taiwan. The NHIRD was launched in 1995 and there are approximately 27.38 million individuals involved in this registry, which enrolls more than 99% of the total population in Taiwan nowadays. All the registrations and claims data of the 1 million beneficiaries are contained in the database, including laboratory and diagnostic tests, drug prescriptions, ambulatory and inpatient care and the International Classification of Disease, Ninth Revision, Clinical Modification (ICD-9-CM) diagnostic codes.

### 2. Population selection

**Fig 1:**
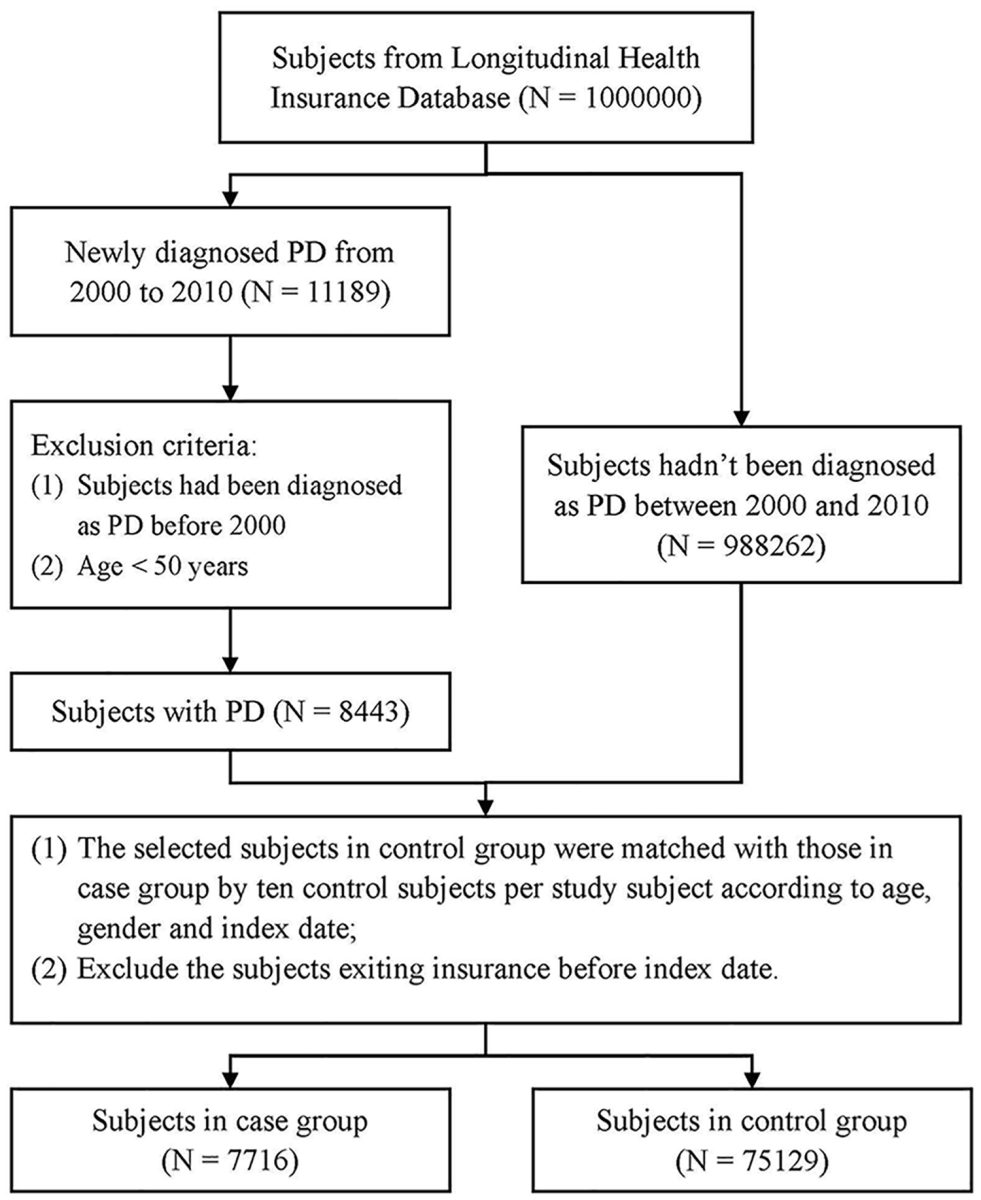
Flowchart of the case-control study.

This population-based case-control study was approved by the Chung Shan Medical University Hospital institutional review board (CSMU No: CS15134). There were two groups enrolled in this study, one was made up from the subjects with newly diagnosed PD and the other was the control group without PD. Subjects with at least twice ambulatory or once admission diagnosis of PD from January 1, 2000 to December 31, 2010 were included in the case group, which contained 11189 of the 1 million beneficiaries. The diagnosis and code (ICD-9-CM 332) were made by neurologists according to the Parkinson’s Disease Society Brain Bank Clinical Diagnostic Criteria [22]. We excluded the subjects with diagnosis of PD before 2000 and age younger than 50 years old, resulting in 8443 in the Parkinson’s disease group. The flowchart of this study was shown in Fig 1.

The control group was derived from the remain beneficiaries who had never been diagnosed of PD between 2000 and 2010 in LHID. The selected subjects in control group were matched with those in case group by ten control subjects per study subject according to age, gender and index date. Finally, we got 7716 subjects in case group and 75129 subjects in control group.

### 3. Baseline variables

The baseline variables of demography were age and gender. Other diseases and affecting factors diagnosed before the diagnosis of PD were also observed, and enrolled with at least twice ambulatory or once inpatient diagnosis, such as RA (ICD-9-CM 714.0), ankylosing spondylitis (ICD-9-CM 720.2), SS (ICD-9-CM 710.2), SLE (ICD-9-CM 710.0), psoriasis (ICD-9-CM 696), osteoarthritis (ICD-9-CM 715), diabetes (ICD-9-CM 250, 357.2, 362.0, 366.41), hypertension (ICD-9-CM 362.11, 401–405, 437.31), atrial fibrillation (ICD-9-CM 427.31), end-stage renal disease (ICD-9-CM 585), dyslipidemia (ICD-9-CM 272) and vasculitis (ICD-9-CM 447.6, 437.4). The diagnosis and code (ICD-9-CM 710.2) were made by rheumatology according to the European classification criteria for Sjögren's syndrome of 2002 [19]. The data collection of the diseases or affect factor all before the index date.

### 4. Statistical analysis

In order to compare the demographic characteristics and affecting factors between case group and control group, Fisher’s exact test was applied for continuous variables, and Pearson’s chi-squared test was applied for categorical variables. The significant factors were then used to obtain the odds ratios (ORs) and 95% confidence intervals (CIs) by performing conditional logistic regression. The crude ORs and 95% CIs would firstly be determined for univariate analysis, and then the adjusted ORs and 95% CIs would be obtained after adjusting other affecting factors which would be considered as confounding variables. All the Statistical analysis was performed by utilizing the SPSS 18 (SPSS Inc., Chicago, IL, USA). Statistical significance was defined as a p value of less than 0.05.

## Results

After matching the non-PD subjects with the PD subjects, there was no significant differences found in age (p = 0.23) and gender (p = 0.76). The mean age of 7716 PD subjects was 72.8 ± 9.0 years, including 3898 (50.5%) women and 3818 (49.5%) men. And the mean age of 75129 non-PD subjects was 72.7 ± 9.0 years, including 38094 (50.7%) women and 37035 (49.3%) men.

The difference on baseline variables between case group and control group was shown in Table 1. 143 of the 7716 PD subjects had SS, which suggested a significant difference as compared with the control group (p < 0.001). In addition, the subjects in study group were more likely to have comorbid ankylosing spondylitis (p = 0.009), osteoarthritis (p < 0.001), diabetes (p < 0.001), hypertension (p < 0.001), atrial fibrillation (p < 0.001), end-stage renal disease (p < 0.001), dyslipidemia (p < 0.001) and vasculitis (p = 0.001).

**Table 1:** Demographic characteristics and affecting factors in the group with PD and the control group.

Characteristics		Parkinson’s disease		Control		
		N = 7716		N = 75129		
	N	n	%	n	%	p-value
Gender						0.76
Female	41992	3898	50.5	38094	50.7	
Male	40853	3818	49.5	37035	49.3	
Age, mean (SD)		72.8 (9.0)		72.7 (9.0)		0.23
Sjogren’s syndrome	1036	143	1.9	893	1.2	< 0.001
Ankylosing spondylitis	496	63	0.8	433	0.6	0.009
RA ^a^	1373	142	1.8	1231	1.6	0.19
SLE ^b^	195	22	0.3	173	0.2	0.34
Psoriasis	953	104	1.3	849	1.1	0.09
Osteoarthritis	33683	3896	50.5	29787	39.6	< 0.001
Diabetes	21499	2680	34.7	18819	25.0	< 0.001
Hypertension	48934	5560	72.1	43374	57.7	< 0.001
Atrial fibrillation	3022	383	5.0	2639	3.5	< 0.001
ESRD ^c^	3670	488	6.3	3182	4.2	< 0.001
Dyslipidemia	23281	2668	34.6	20613	27.4	< 0.001
Vasculitis	684	88	1.1	596	0.8	0.001

^a^ Rheumatoid arthritis,

^b^ Systemic Lupus Erythematosus,

^c^ End-stage renal disease

**Fig 2:**
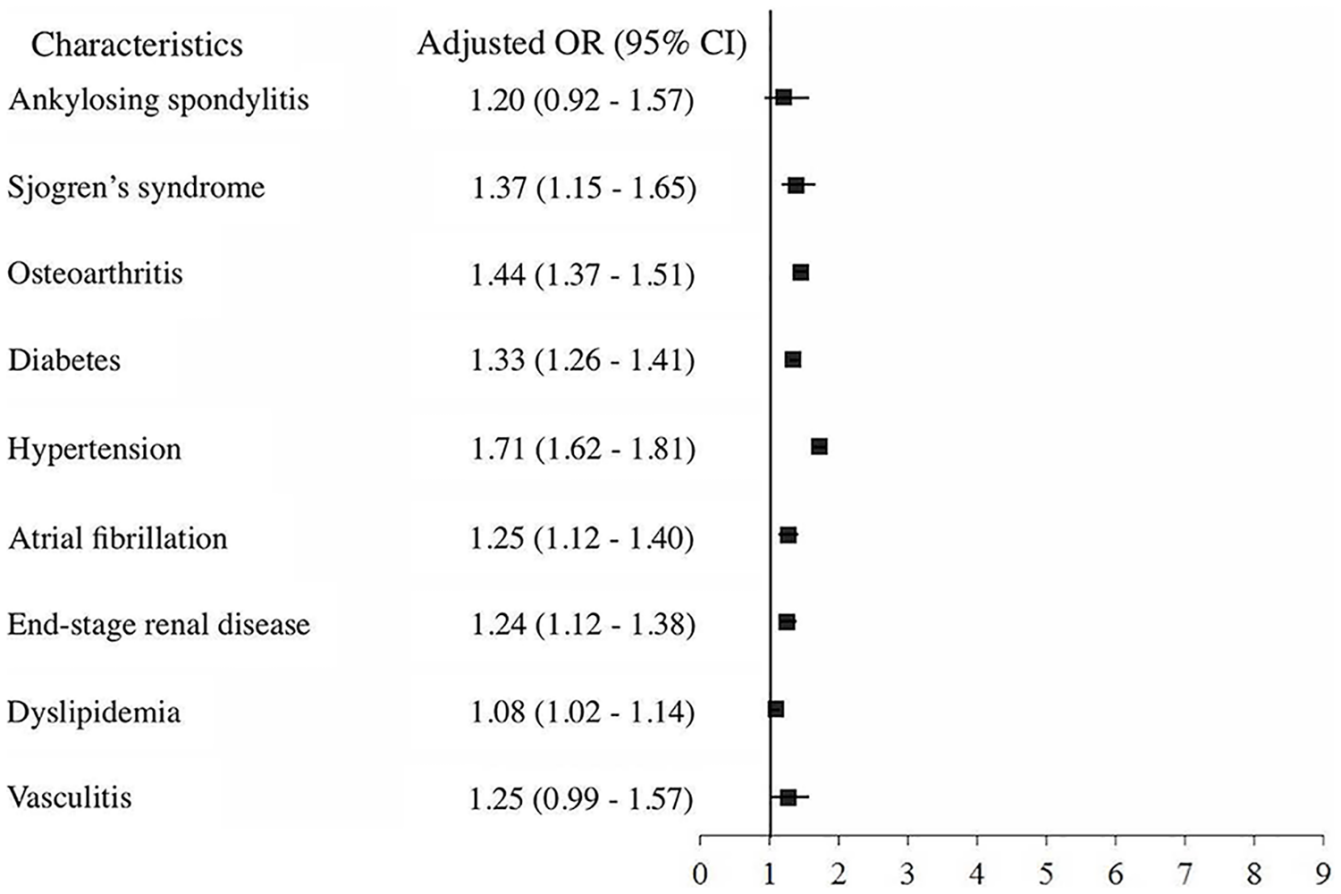
The forest plots of adjusted OR with 95% CI for the Sjogren’s syndrome (OR = 1.37, 95% CI = 1.15–1.65, p < 0.001).

The association between affecting factors and the risk of PD was illustrated in Table 2. For univariate analysis, SS was significantly associated with increased risk of PD (OR, 1.56; 95% CI 1.30–1.86, p < 0.01). On the other hand, ankylosing spondylitis (OR, 1.41; 95% CI 1.08–1.84, p < 0.05), osteoarthritis (OR, 1.60; 95% CI 1.53–1.69, p < 0.01), diabetes (OR, 1.61; 95% CI 1.53–1.69, p < 0.01), hypertension (OR, 1.99; 95% CI 1.89–2.10, p < 0.01), atrial fibrillation (OR, 1.42; 95% CI 1.27–1.59, p < 0.01), end-stage renal disease (OR, 1.52; 95% CI 1.38–1.68, p < 0.01), dyslipidemia (OR, 1.42; 95% CI 1.35–1.50, p < 0.01) and vasculitis (OR, 1.44; 95% CI 1.15–1.80, p < 0.01) were also related to the development of PD. After adjusting confounding variables, the adjusted OR of SS was 1.37 (95% CI 1.15–1.64, p < 0.01), which indicated that SS might be associated with the increased risk for developing PD. Meanwhile, the adjusted ORs of other factors were also shown in Table 2. Except ankylosing spondylitis and vasculitis, other factors mentioned above were also related to the PD. The Forest plots for adjusted ORs were shown in Fig 2.

**Table 2:** Risk factors for PD.

Variables	Crude OR	95% CI		Adjusted OR	95% CI	
		Lower	Upper		Lower	Upper
Sjogren’s syndrome	1.56**	1.30	1.86	1.38**	1.15	1.66
Ankylosing spondylitis	1.41*	1.08	1.84	1.20	0.92	1.57
Osteoarthritis	1.60**	1.53	1.69	1.44**	1.37	1.51
Diabetes	1.61**	1.53	1.69	1.33**	1.26	1.41
Hypertension	1.99**	1.89	2.10	1.71**	1.62	1.81
Atrial fibrillation	1.42**	1.27	1.59	1.25**	1.12	1.40
ESRD ^a^	1.52**	1.38	1.68	1.24**	1.12	1.38
Dyslipidemia	1.42**	1.35	1.50	1.08**	1.02	1.14
Vasculitis	1.44**	1.15	1.80	1.25	0.99	1.57

^a^ End-stage renal disease,

* p < 0.05,

** p < 0.01

## Discussion

In this study, SS was proposed as a risk factor for the development of PD. To the best of our knowledge, this is the first study to apply a nationwide longitudinal population-based database to estimate the relationship between SS and PD.

Despite of extensive studies on PD, its underlying cause and pathogenesis still remains unknown. Complex interactions between environmental and multiple genetic factors has been shown to contribute to dopaminergic neuronal cell loss and parkinsonism, which would final establish the disease. In this study, the number of patients with history of SS in subjects who experienced PD was significantly higher than that in subjects who did not (p <0.001). In addition, the adjusted OR for PD in patients with SS was 1.37 (95% CI 1.15–1.66, p < 0.01) as compared with that who without. Considering these results, it could be suggested that SS was a risk affecting factor for PD.

Several studies have reported that SS was associated with PD, and their results were well correlated with ours [17,18]. Our study involved a total of 143 patients who were suffering from both SS and PD. The slight large sample size may offer a good representation of ethnic Asian patients, and provide more information in approaching the connection between SS and PD.

SS is known as a disease with mononuclear lymphocytic infiltration of salivary and lacrimal glands, and it would also have a extraglandular involvement of other organs, such as lung, liver, kidney and nervous system. The most well-recognized pathogenesis is the immunologically mediated mechanisms. Autoantibodies are usually detected in patients with SS, and play an important role in connecting to connective tissue disorders. Anti-SSA and anti-SSB could be respectively found in 63% and 40% patients with SS, and they would be associated with the involvement of CNS diseases [23]. In addition, some researchers enrolled SS patients who suffered from peripheral and central nervous system abnormalities into study, and examined the antineuronal antibodies in their plasma. The results showed a higher present of these antibodies in patients with major neurologic complications than those without [24]. An immunologically mediated mechanism between SS and PD could be conjectured, in which antibodies from SS patients may directly damage the basal ganglia and contribute to PD.

Hassin-Baer S et al. [25] examined the serum of patients with both SS and PD, and found high titers of anti-beta2-glycoprotein IgG, which is an autoantibody strongly associated with anti-cardiolipin antibodies (aCL), antiphospholipid syndrome, and thromboembolic phenomena. Several previous studies have introduced that there would be a connection between aCL antibodies and PD [26–28]. Moreover, some evidences of CNS vascular lesions have been found in a proportion of cases of SS with neurologic involvement [23]. It was suggested that there might be an underlying vascular mechanism for the development of PD [25]. The role of antibodies in the pathogenesis of PD in SS is still unclear, and further studies is required. For our study, more analysis for the subjects, such as antibodies in their serum, is needed to assess the underlying mechanism and detail role of antibodies in relations between SS and PD.

There are two neurotransmitter systems in CNS, one is dopaminergic system and another is cholinergic system. Only a coordination of these systems could promise a normal physiological activity. PD primarily results from the death of dopaminergic neurons in the substantia nigra, which would break the balance between dopaminergic activity and cholinergic activity, and cause a variety of disorders. Anticholinergic drugs are commonly used to reduce cholinergic activity, and treat the symptoms of PD [29]. Oppositely, for the purpose of increasing the salivary secretion and relieving the dry symptom, cholinergic agonists are usually applied to treat the SS [30,31]. It might be conjectured that the application of drugs in treating SS would affect the development of PD, and aggravate the symptoms of PD. This assumption provides an underlying pharmacology mechanism among SS and PD, and further prospective studies are needed to warrant it.

In generally speaking, the pathogenesis of PD is multifactorial. As well correlated with previous studies [7–10,14,15], our study showed diabetes, hypertension, end-stage renal disease, dyslipidemia, atrial fibrillation and osteoarthritis were significantly connected with PD. Other than some studies [11,12], RA, SLE and psoriasis were not significant associated with PD in this study, it might be owing to the difference on sample size and ethnicity. Ankylosing spondylitis and vasculitis were not significantly related to PD after adjusting the confounding variables, it was possible due to the relative small sample size as compared with other variables. Further investigation with more sample size and other ethnic population would be needed.

Most of the PD would occur after 50 years old [5], which was similar to the demographics of our newly diagnosed PD population in terms of age. Some studies suggested that age and gender might be associated with PD [32,33], Yet, the age and gender were not significant affecting factors for PD in this study. The possible reason for this discrepancy between previous studies and ours may be the match of age and gender for a case-control design of our study.

There are some possible limitations to the current study. First, this is a retrospective study, and the design of this study might cause a potential risk of selection bias, especially in the portion of patients. Second, the study used the ICD codes from the National Health Insurance claim database, and could be affected by the diagnostic accuracy of the database. Third, information on individual behaviors, such as smoking and alcohol consumption, and the subtypes of SS and PD are unavailable from the database. Fourth, the study results are come from the Eastern population, and they may need to be validated in study for other ethnic population. Finally, this is a case-control study, and the effect of SS on PD over time could not be estimated, a cohort study between them is required in the future.

## Conclusion

This population-based case-control study preliminarily proposed that SS was significant associated with an increased risk of PD. The pathophysiology and causality need to be further investigated in bench works or randomized clinical trials.

## Supporting information

S1 Table Operational definitions of variables. (DOCX)
