# Supplementary material for: Impact of Sjogren's syndrome on Parkinson’s disease: A nationwide case-control study
Source: PLoS One. 2017 Jul 13;12(7):e0175836. doi: 10.1371/journal.pone.0175836 (PMC5509109; doi:10.1371/journal.pone.0175836)
Supplement: S1 Table — (DOCX) [file pone.0175836.s001.docx]

| **S1 Table. Operational definitions of variables** | |
| --- | --- |
| **Variable** | **Operational Definition** |
| **Dependent variable** | |
| Parkinson's disease | Diagnosis of Parkinson's disease (ICD-9-CM=332) at least twice ambulatory or once inpatient from 2000 to 2010. |
| Control | No diagnosis of Parkinson's disease between 2000 and 2010 |
| Index date | The date of earliest diagnosis of Parkinson's disease between 2000 and 2010 |
| **Independent variable** | |
| Year of the data | 2000/1/1~2010/12/31 |
| Gender | Male/Female |
| Age | Index date |
| Rheumatoid arthritis | Diagnosis of Rheumatoid arthritis (ICD-9-CM=714.0) at least twice ambulatory or once inpatient before index date. |
| Ankylosing spondylitis | Diagnosis of Ankylosing spondylitis (ICD-9-CM=720.2) at least twice ambulatory or once inpatient before index date. |
| Sjogren's syndrome | Diagnosis of Sjogren's syndrome (ICD-9-CM=710.2) at least twice ambulatory or once inpatient before index date. |
| SLE | Diagnosis of Systemic Lupus Erythematosus (ICD-9-CM=710.0) at least twice ambulatory or once inpatient before index date. |
| Psoriasis | Diagnosis of Psoriasis (ICD-9-CM=696) at least twice ambulatory or once inpatient before index date. |
| Osteoarthritis | Diagnosis of Psoriasis (ICD-9-CM=715) at least twice ambulatory or once inpatient before index date. |
| Diabetes | Diagnosis of Diabetes (ICD-9-CM=250, 357.2, 362.0, 366.41) at least twice ambulatory or once inpatient before index date. |
| Hypertension | Diagnosis of Hypertension (ICD-9-CM=362.11, 401-405, 437.2) at least twice ambulatory or once inpatient before index date. |
| Atrial fibrillation | Diagnosis of Atrial fibrillation (ICD-9-CM=427.31) at least twice ambulatory or once inpatient before index date. |
| End-stage renal disease | Diagnosis of ESRD (ICD-9-CM=585) at least twice ambulatory or once inpatient before index date. |
| Dyslipidemia | Diagnosis of Dyslipidemia (ICD-9-CM=272) at least twice ambulatory or once inpatient before index date. |
| Vasculitis | Diagnosis of Vasculitis (ICD-9-CM=447.6, 437.4) at least twice ambulatory or once inpatient before index date. |
